# Supplementary material for: Targeted cortical reorganization using optogenetics in non-human primates
Source: eLife. 2018 May 29;7:e31034. doi: 10.7554/eLife.31034 (PMC5986269; doi:10.7554/eLife.31034)
Supplement: Figure 3—source code 1. [file elife-31034-fig3-code1.zip › helpers/kakearney-boundedline-pkg-32f2a1f/Inpaint_nans/demo/html/inpaint_nans_demo.html]

inpaint\_nans\_demo


 


```
% Surface fit artifact removal
[x,y] = meshgrid(0:.01:1);
z0 = exp(x+y);

znan = z0;
znan(20:50,40:70) = NaN;
znan(30:90,5:10) = NaN;
znan(70:75,40:90) = NaN;

z = inpaint_nans(znan,3);

% Comparison to griddata
k = isnan(znan);
zk = griddata(x(~k),y(~k),z(~k),x(k),y(k));
zg = znan;
zg(k) = zk;

close all
figure
surf(z0)
title 'Original surface'

figure
surf(znan)
title 'Artifacts (large holes) in surface'

figure
surf(zg)
title(['Griddata inpainting (',num2str(sum(isnan(zg(:)))),' NaNs remain)'])

figure
surf(z)
title 'Inpainted surface'

figure
surf(zg-z0)
title 'Griddata error surface'

figure
surf(z-z0)
title 'Inpainting error surface (Note z-axis scale)'
```

     

Published with MATLAB® 7.0.1
