# Supplementary material for: Targeted cortical reorganization using optogenetics in non-human primates
Source: eLife. 2018 May 29;7:e31034. doi: 10.7554/eLife.31034 (PMC5986269; doi:10.7554/eLife.31034)
Supplement: Figure 3—source code 1. [file elife-31034-fig3-code1.zip › helpers/kakearney-boundedline-pkg-32f2a1f/Inpaint_nans/doc/Nomination comments.rtf]

Nomination comments:Inpaint_nans fills a hole in matlab. (Yes, the pun was intentional.) But thereis indeed a niche that inpaint_nans falls into.The alternative to inpaint_nans is griddata (interp1 can be used for the 1-d problems) but griddata fails to extrapolate well. Griddata also has seriousproblems when its data already lies on a grid, due to its use of a Delaunay triangulation. The other serious problem with the use of griddata is thetriangulation itself. The shape of the hole to be filled can sometimes resultin triangles with a poor aspect ratio (long, thin triangles) which are in turnpoor for interpolation. In fact, Griddata can even leave interior pointsuninterpolated (see the tests.)A future plan for inpaint_nans is to add an option that will use a locallyanisotropic membrane model. This will allow better modeling for certainclasses of wavy surfaces. I'm also highly tempted to remove method 5.I've never really liked it, having put it in at the request of one user. It hasno valid theory behind it in the context of inpaint_nans.In the interest of openness, I'll also say what inpaint_nans does not do. Itdoes not handle non-uniform grids. It is limited by the amount of memory in the size of the arrays it can handle, although some of the methods wereexplicitly provided to be more memory efficient than others. Inpaint_nansalso makes heavy use of sparse matrices, so surprisingly large problemsare accessible.Finally, while inpaint_nans does work for 1-d problems, they are not mytarget. Interp1 (with 'spline' as the method) is as accurate, and should befaster in general.John
