# Supplementary material for: Targeted cortical reorganization using optogenetics in non-human primates
Source: eLife. 2018 May 29;7:e31034. doi: 10.7554/eLife.31034 (PMC5986269; doi:10.7554/eLife.31034)
Supplement: Figure 3—source code 1. [file elife-31034-fig3-code1.zip › README.rtf]

Figure3B_SourceDataContains 6 variables:The following two variables are from one example session.C_ex1 - 	each element of this vector contains the theta coherence [4-8Hz] between the stimulation channel and a secondary channel ER_ex1 - each element of this vector contains the stimulus evoked response ratio between the stimulation channel and a secondary channelThe following two variables are from another example session.C_ex2 - 	each element of this vector contains the theta coherence [4-8Hz] between the stimulation channel and a secondary channel ER_ex2 - each element of this vector contains the stimulus evoked response ratio between the stimulation channel and a secondary channelThe following two variables are from a third example session.C_ex3 - 	each element of this vector contains the theta coherence [4-8Hz] between the stimulation channel and a secondary channel ER_ex3 - each element of this vector contains the stimulus evoked response ratio between the stimulation channel and a secondary channelFigure3C_SourceDataContains 4 variables:blocks = [1,6], corresponding to the first and final recording and test blocks analyzed in each experimentfreqs - matrix, each row is a frequency band used for coherence measurements in 'C'C - cell array {sessions x blocks}	each cell contains a matrix [secondary channels x frequencies] 	each element of this matrix contains the coherence between the stimulation channel and a secondary channel at a frequency band corresponding to the frequencies in the matrix 'freqs'ER - cell array {sessions x blocks}	each cell contains a vector (secondary channels x 1)	each element of this vector contains the stimulus evoked response ratio between the stimulation channel and a secondary channel
