# Supplementary figures and images for: Targeted cortical reorganization using optogenetics in non-human primates
Source: eLife. 2018 May 29;7:e31034. doi: 10.7554/eLife.31034 (PMC5986269; doi:10.7554/eLife.31034)

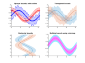

Supplement: Figure 3—source code 1. [file elife-31034-fig3-code1.zip › helpers/kakearney-boundedline-pkg-32f2a1f/boundedline_readme.png]

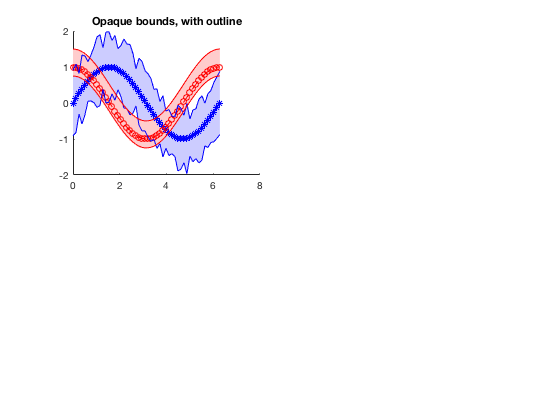

Supplement: Figure 3—source code 1. [file elife-31034-fig3-code1.zip › helpers/kakearney-boundedline-pkg-32f2a1f/boundedline_readme_01.png]

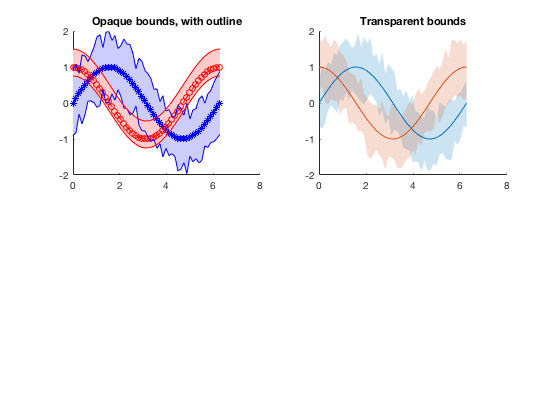

Supplement: Figure 3—source code 1. [file elife-31034-fig3-code1.zip › helpers/kakearney-boundedline-pkg-32f2a1f/boundedline_readme_02.png]

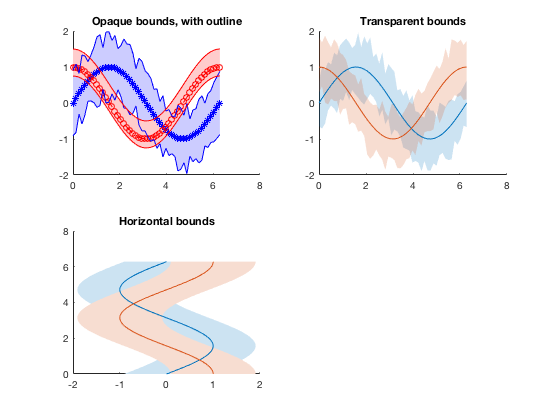

Supplement: Figure 3—source code 1. [file elife-31034-fig3-code1.zip › helpers/kakearney-boundedline-pkg-32f2a1f/boundedline_readme_03.png]

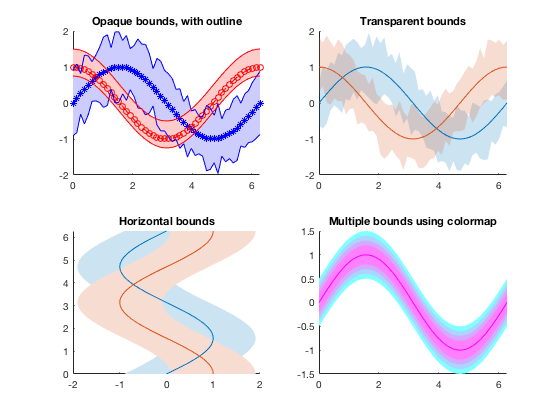

Supplement: Figure 3—source code 1. [file elife-31034-fig3-code1.zip › helpers/kakearney-boundedline-pkg-32f2a1f/boundedline_readme_04.png]

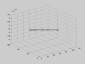

Supplement: Figure 3—source code 1. [file elife-31034-fig3-code1.zip › helpers/kakearney-boundedline-pkg-32f2a1f/Inpaint_nans/demo/html/inpaint_nans_demo.png]

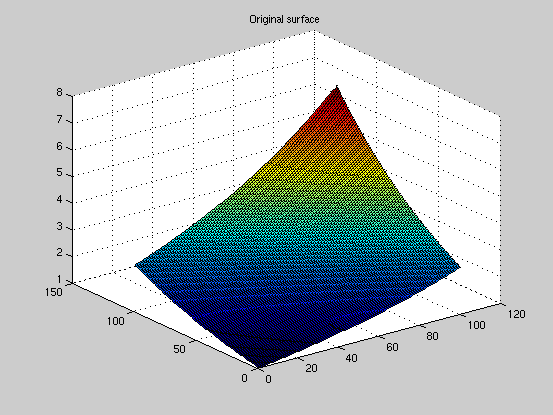

Supplement: Figure 3—source code 1. [file elife-31034-fig3-code1.zip › helpers/kakearney-boundedline-pkg-32f2a1f/Inpaint_nans/demo/html/inpaint_nans_demo_01.png]

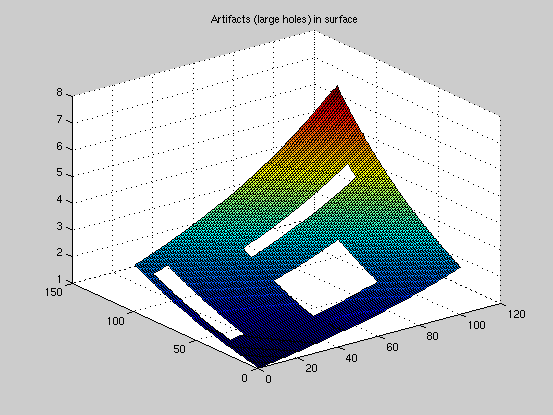

Supplement: Figure 3—source code 1. [file elife-31034-fig3-code1.zip › helpers/kakearney-boundedline-pkg-32f2a1f/Inpaint_nans/demo/html/inpaint_nans_demo_02.png]

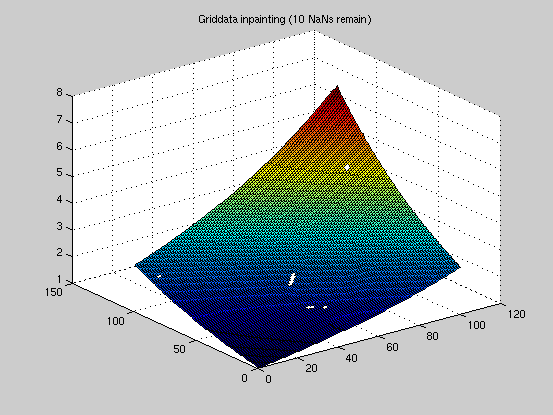

Supplement: Figure 3—source code 1. [file elife-31034-fig3-code1.zip › helpers/kakearney-boundedline-pkg-32f2a1f/Inpaint_nans/demo/html/inpaint_nans_demo_03.png]

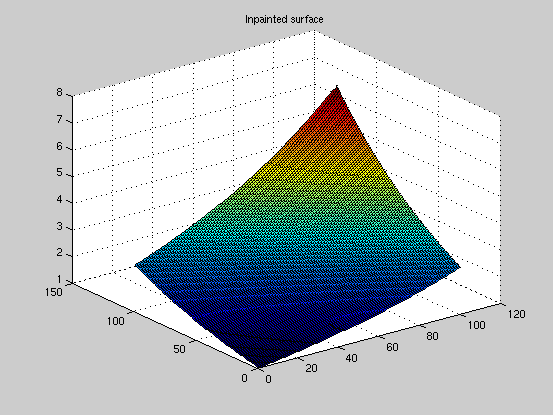

Supplement: Figure 3—source code 1. [file elife-31034-fig3-code1.zip › helpers/kakearney-boundedline-pkg-32f2a1f/Inpaint_nans/demo/html/inpaint_nans_demo_04.png]

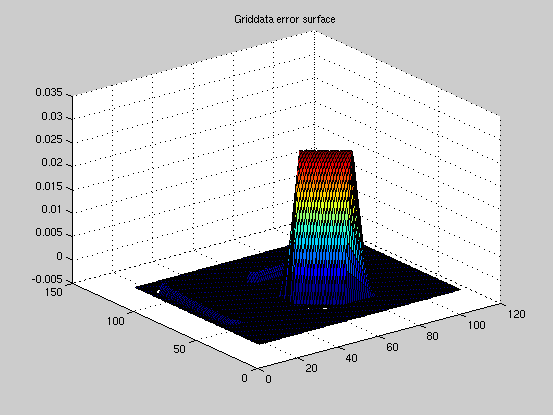

Supplement: Figure 3—source code 1. [file elife-31034-fig3-code1.zip › helpers/kakearney-boundedline-pkg-32f2a1f/Inpaint_nans/demo/html/inpaint_nans_demo_05.png]

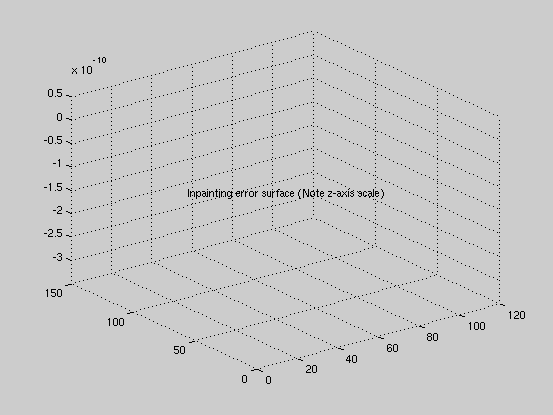

Supplement: Figure 3—source code 1. [file elife-31034-fig3-code1.zip › helpers/kakearney-boundedline-pkg-32f2a1f/Inpaint_nans/demo/html/inpaint_nans_demo_06.png]

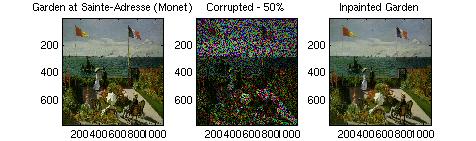

Supplement: Figure 3—source code 1. [file elife-31034-fig3-code1.zip › helpers/kakearney-boundedline-pkg-32f2a1f/Inpaint_nans/garden50.jpg]

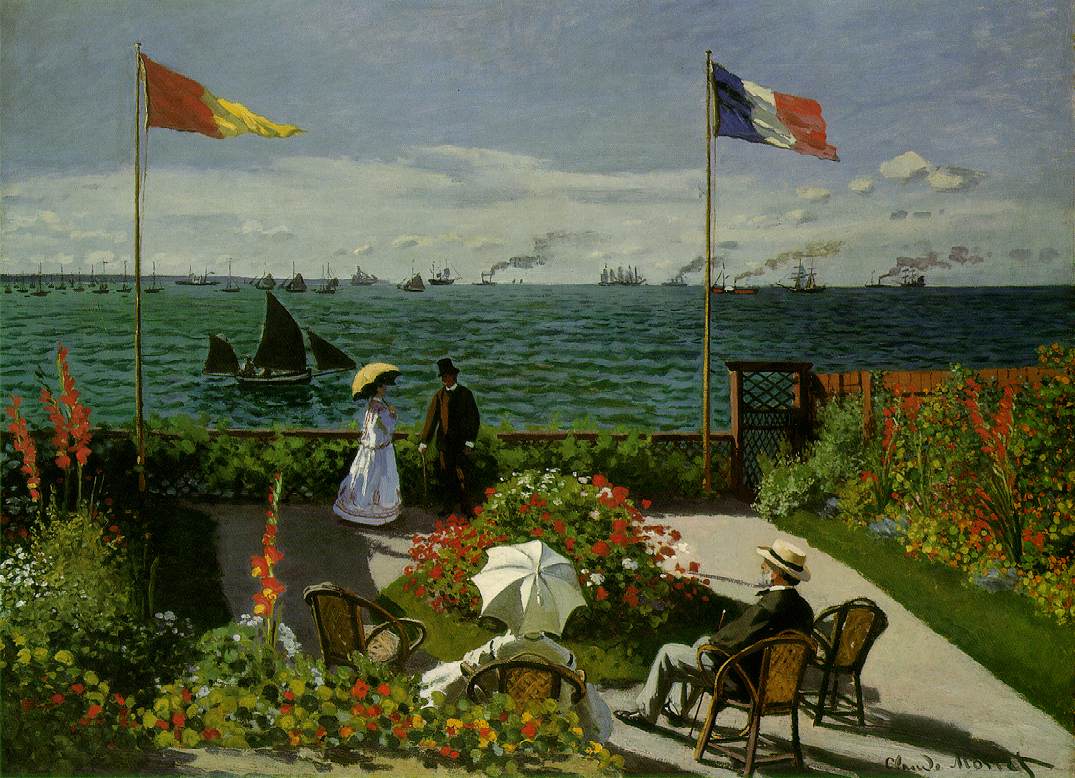

Supplement: Figure 3—source code 1. [file elife-31034-fig3-code1.zip › helpers/kakearney-boundedline-pkg-32f2a1f/Inpaint_nans/monet_adresse.jpg]
