# Supplementary material for: Targeted cortical reorganization using optogenetics in non-human primates
Source: eLife. 2018 May 29;7:e31034. doi: 10.7554/eLife.31034 (PMC5986269; doi:10.7554/eLife.31034)
Supplement: Figure 4—source code 1. [file elife-31034-fig4-code1.zip › README.rtf]

Figure4_SourceDataFigure4B-C_SourceDataContains 5 variables:pk2tr - cell array, each cell contains the evoked response ratio (ERR) for all secondary channels. Column1 is the ERR for block1 (initial). Column2 is the ERR for block6 (final).S - cell array, each cell contains the metadata for an experiment corresponding to the same cell in 'pk2tr'secondary - cell array, each cell contains the secondary channels for the examples plotted in 4Bstim_ch - cell array, each cell contains the stimulation channels for the examples plotted in 4Bbad_channels - vector of bad channels for the examples plotted in 4BFigure4D_SourceDataContains 6 variablespktr1_none - vector of session averages (across channels) of initial evoked response ratio (ERR). Only contains sessions in which laser was not active during conditioning.pktr2_none - vector of session averages (across channels) of final evoked response ratio (ERR). Only contains sessions in which laser was not active during conditioning.pktr1M1 - vector of session averages (across channels) of initial evoked response ratio (ERR). Only contains sessions in which laser was positioned in M1.pktr2M1 - vector of session averages (across channels) of final evoked response ratio (ERR). Only contains sessions in which laser was positioned in M1.pktr1S1 - vector of session averages (across channels) of initial evoked response ratio (ERR). Only contains sessions in which laser was positioned in S1.pktr2S1 - vector of session averages (across channels) of final evoked response ratio (ERR). Only contains sessions in which laser was positioned in S1.Figure4E-F_SourceDataContains 6 variables:C0_M1S1 - cell array, where each cell is a difference session. Each cell contains a vector of initial theta coherence values between the stim channel and secondary channels (in other area).Cf_M1S1 - cell array, where each cell is a difference session. Each cell contains a vector of final theta coherence values between the stim channel and secondary channels (in other area).S - cell array, each cell contains the metadata for an experiment corresponding to the same cell in 'CX_M1S1'secondary - cell array, each cell contains the secondary channels for the examples plotted in 4Bstim_ch - cell array, each cell contains the stimulation channels for the examples plotted in 4Bbad_channels - vector of bad channels for the examples plotted in 4BFigure4G_SourceDataContains 4 variables:C0_M1S1 - cell array {3 x 15}, where each cell is a difference session. The first row in the cell array contains a cell array of control sessions in which the laser was inactive. The second row contains a cell array of sessions in which the delay between the lasers during conditioning was long (70ms or 100ms) or only one laser was active. The third row contains a cell array of sessions in which the delay between the lasers during conditioning was short (10ms or 30ms). Each cell contains a vector of initial theta coherence values between the stim channel and secondary channels (in other area).Cf_M1S1 - cell array {3 x 15}, where each cell is a difference session. The first row in the cell array contains a cell array of control sessions in which the laser was inactive. The second row contains a cell array of sessions in which the delay between the lasers during conditioning was long (70ms or 100ms) or only one laser was active. The third row contains a cell array of sessions in which the delay between the lasers during conditioning was short (10ms or 30ms). Each cell contains a vector of final theta coherence values between the stim channel and secondary channels (in other area).freqs - matrix, where each row corresponds to a frequency band that was used.NAMES - cell array, where each cell contains the name of the animal used in the corresponding experiment.Figure4H_SourceDataContains 4 variables:blocks = [1,6], corresponding to the first and final recording and test blocks analyzed in each experimentfreqs - matrix, each row is a frequency band used for coherence measurements in 'C'C - cell array {sessions x blocks}	each cell contains a matrix [secondary channels x frequencies] 	each element of this matrix contains the coherence between the stimulation channel 	and a secondary channel at a frequency band corresponding to the frequencies in the 	matrix 'freqs'ER - cell array {sessions x blocks}	each cell contains a vector (secondary channels x 1)	each element of this vector contains the evoked response ratio between the 	stimulation channel and a secondary channelslopes_all - cell array { stim_sessions _block1, stim_sessions_block6 ; no_stim_block1, no_stim_block6 }	each cell contains a matrix [num_sessions x 15freqbands]	each element contains a fit regression slope for a particular example session/blockfreq_bands - frequency bands corresponding to slopes_all
