# Supplementary material for: Targeted cortical reorganization using optogenetics in non-human primates
Source: eLife. 2018 May 29;7:e31034. doi: 10.7554/eLife.31034 (PMC5986269; doi:10.7554/eLife.31034)
Supplement: Figure 4—figure supplement 1—source code 1. [file elife-31034-fig4-figsupp1-code1.zip › Figure4-FigureSupplement1-README.rtf]

Figure4_FigureSupplement1_SourceDataFigure4_FigureSupplement1_SourceDataContains 4 variables:blocks = [1,6], corresponding to the first and final recording and test blocks analyzed in each experimentfreqs - matrix, each row is a frequency band used for coherence measurements in 'C'C - cell array {sessions x blocks}	each cell contains a matrix [secondary channels x frequencies] 	each element of this matrix contains the coherence between the stimulation channel 	and a secondary channel at a frequency band corresponding to the frequencies in the 	matrix 'freqs'ER - cell array {sessions x blocks}	each cell contains a vector (secondary channels x 1)	each element of this vector contains the evoked response ratio between the stimulation channel and a secondary channel
