# Supplementary material for: Targeted cortical reorganization using optogenetics in non-human primates
Source: eLife. 2018 May 29;7:e31034. doi: 10.7554/eLife.31034 (PMC5986269; doi:10.7554/eLife.31034)
Supplement: Figure 4—figure supplement 2—source code 1. [file elife-31034-fig4-figsupp2-code1.zip › Figure4-FigureSupplement2-README.rtf]

Figure4_FigureSupplement2_SourceDataFigure4_FigureSupplement2_SourceDataContains 8 variablesfreqs - frequency bands used in analysisP0 - cell array {experimental condtions x num freq bands}  (experimental conditions are - ‘no stim’, ‘long latency stim’ and ‘short latency stim’)	contains the normalized power (power on secondary channels normalized by power at stim site) at beginning of each sessionPf - cell array {experimental condtions x num freq bands}  (experimental conditions are - ‘no stim’, ‘long latency stim’ and ‘short latency stim’)	contains the normalized power (power on secondary channels normalized by power at stim site) at end of each sessionP0_sec - cell array {experimental condtions x num freq bands}  (experimental conditions are - ‘no stim’, ‘long latency stim’ and ‘short latency stim’)	contains the power on secondary channels at beginning of each sessionPf_sec - cell array {experimental condtions x num freq bands}  (experimental conditions are - ‘no stim’, ‘long latency stim’ and ‘short latency stim’)	contains the power on secondary channels at end of each sessionP0_stim - cell array {experimental condtions x num freq bands}  (experimental conditions are - ‘no stim’, ‘long latency stim’ and ‘short latency stim’)	contains the power at stim site at beginning of each sessionPf_stim - cell array {experimental condtions x num freq bands}  (experimental conditions are - ‘no stim’, ‘long latency stim’ and ‘short latency stim’)	contains the power at stim site at end of each session
