# Supplementary material for: Targeted cortical reorganization using optogenetics in non-human primates
Source: eLife. 2018 May 29;7:e31034. doi: 10.7554/eLife.31034 (PMC5986269; doi:10.7554/eLife.31034)
Supplement: Figure 5—source code 1. [file elife-31034-fig5-code1.zip › Figure5README.rtf]

Figure5_SourceData

Contains 5 variables:
blocks = [1,2,3,4,5,6], corresponding to the recording and test blocks analyzed in each experiment
C - cell array {monkey x blocks}
	each cell contains a cell array of sessions. Each session contains a vector of the 	theta coherence between the stimulation channel and the secondary channels 
test_pk2tr - cell array {monkey x blocks}
	each cell contains a cell array of sessions. Each session contains a vector of the 		evoked response ratio between the stimulation channel and the secondary channels 
cond_pk2tr - cell array {monkey x blocks}
	each cell contains a cell array of sessions. Each session contains a matrix of the 		evoked response ratio of 100 laser pulses between the stimulation channel and the 	secondary channels. Note that the first row corresponds to the first 100 laser pulses 	and so on. Also note that there are only 5 conditioning blocks so cond_pk2tr is smaller 	than 'C' and 'test_pk2tr'
S - cell array containing metadata for each session. Note that this does not repeat the 	metadata for each block.
