# Supplementary material for: Targeted cortical reorganization using optogenetics in non-human primates
Source: eLife. 2018 May 29;7:e31034. doi: 10.7554/eLife.31034 (PMC5986269; doi:10.7554/eLife.31034)
Supplement: Figure 7—figure supplement 1—source code 1. [file elife-31034-fig7-figsupp1-code1.zip › README.rtf]

Figure7_FigureSupplement1A-B_SourceDataContains 5 variables:C0_1 - (96ch x 96ch) matrix of pairwise theta coherence values for an example session. The coherence values were calculated for a mutually exclusive subset of the data in an example recording block.C0_2 - (96ch x 96ch) matrix of pairwise theta coherence values for an example session. The coherence values were calculated for a mutually exclusive subset of the data in an example recording block.C2 - (96ch x 96ch) matrix of pairwise theta coherence values for an example session. The coherence values were calculated during the conditioning block immediately after the example block in 'C0_1' and 'C0_2'.Cf - (96ch x 96ch) matrix of pairwise theta coherence values for an example session. The coherence values were calculated for a subset of the data in the recording block immediately after the conditioning block in 'C2'.L - cell array, each cell contains the channel closest to where a laser was positioned in this example experiment.
