# Supplementary material for: Targeted cortical reorganization using optogenetics in non-human primates
Source: eLife. 2018 May 29;7:e31034. doi: 10.7554/eLife.31034 (PMC5986269; doi:10.7554/eLife.31034)
Supplement: Figure 7—figure supplement 2—source code 1. [file elife-31034-fig7-figsupp2-code1.zip › Figure7-FigureSupplement2-README.rtf]

Figure7_FigureSupplement2_SourceDataContains 3 variables :The slope, r^2, and p-values were obtained via linear regression btw the change in theta coherence and the stimulus evoked coherence. Each variable is organized by the experimental condition, and then further organized into a matrix [num_sessions x block]slope - struct with fields	.none - for no stim sessions	.long - for single-site and long latency stim sessions	.short - for short stim sessionsr2 - struct with fields	.none - for no stim sessions	.long - for single-site and long latency stim sessions	.short - for short stim sessionsp - struct with fields	.none - for no stim sessions	.long - for single-site and long latency stim sessions	.short - for short stim sessions
