# Supplementary material for: Targeted cortical reorganization using optogenetics in non-human primates
Source: eLife. 2018 May 29;7:e31034. doi: 10.7554/eLife.31034 (PMC5986269; doi:10.7554/eLife.31034)
Supplement: Table 2—source code 1. [file elife-31034-table2-code1.zip › SupplementalTable2_README.rtf]

SupplementalTable2_SourceDataSupplementalTable2_SourceDataContains 31 variables:blocks = [1,6], corresponding to the first and final recording and test blocks analyzed in each experimentfreqs - matrix, each row is a frequency band used for coherence measurements in 'C'C - cell array {sessions x blocks}	each cell contains a matrix [secondary channels x frequencies] 	each element of this matrix contains the coherence between the stimulation channel 	and a secondary channel at a frequency band corresponding to the frequencies in the 	matrix 'freqs'Remaining variables correspond to different measures of the evoked response - broadband amplitude, broadband energy, broadband slope, and high gamma energy. For each measure there are 7 associated variables. For example, broadband amplitude has these 7 associated variablesbroadband_amp - cell array {sessions x blocks}	each cell contains a vector (secondary channels x 1)	each element of this vector contains the evoked response ratio of this measure between the stimulation channel and a secondary channel broadband_amp1_nostim - summary data, average initial value of this measure’s evoked response ratio for each ‘no stim’ sessionbroadband_amp2_nostim - summary data, average final value of this measure’s evoked response ratio for each ‘no stim’ sessionbroadband_amp1_M1stim - summary data, average initial value of this measure’s evoked response ratio for each ‘M1 stim’ sessionbroadband_amp2_M1stim - summary data, average final value of this measure’s evoked response ratio for each ‘M1 stim’ sessionbroadband_amp1_S1stim - summary data, average initial value of this measure’s evoked response ratio for each ‘S1 stim’ sessionbroadband_amp2_S1stim - summary data, average final value of this measure’s evoked response ratio for each ‘S1 stim’ session
