# Supplementary material for: Targeted cortical reorganization using optogenetics in non-human primates
Source: eLife. 2018 May 29;7:e31034. doi: 10.7554/eLife.31034 (PMC5986269; doi:10.7554/eLife.31034)
Supplement: Transparent reporting form [file elife-31034-transrepform.docx]

***eLife’s* transparent reporting form**

We encourage authors to provide detailed information *within their submission* to facilitate the interpretation and replication of experiments. Authors can upload supporting documentation to indicate the use of appropriate reporting guidelines for health-related research (see [EQUATOR Network](http://www.equator-network.org/%20)), life science research (see the [BioSharing Information Resource](https://biosharing.org/)), or the [ARRIVE guidelines](http://www.plosbiology.org/article/info:doi/10.1371/journal.pbio.1000412) for reporting work involving animal research. Where applicable, authors should refer to any relevant reporting standards documents in this form.

If you have any questions, please consult our Journal Policies and/or contact us: [editorial@elifesciences.org](mailto:editorial@elifesciences.org).

**Sample-size estimation**

- You should state whether an appropriate sample size was computed when the study was being designed
- You should state the statistical method of sample size computation and any required assumptions
- If no explicit power analysis was used, you should describe how you decided what sample (replicate) size (number) to use

Please outline where this information can be found within the submission (e.g., sections or figure legends), or explain why this information doesn’t apply to your submission:

We did not use an explicit power analysis when designing our experiments.

We used two rhesus macaques as our experimental subjects. This number does not reflect the outcome of a statistical analysis but rather reflects the standards of the field. The idea is that while statistical tests are performed within-subject, one must make sure that the results are not idiosyncratic to a single animal. By replicating one’s findings in the second animal, this concern is sufficiently alleviated.

Statistical analyses are all performed either across measurements within a session or across sessions. The number of measurements per session was determined by the duration of the experiment, and this was based not on statistical considerations, but on the time we expected it to take for the learning curves to asymptote, based on previously reported learning curves and our own pilot data. The number of sessions conducted per animal was determined by implant viability: over time neural recordings and optical stimulation degrade.

**Replicates**

- You should report how often each experiment was performed
- You should include a definition of biological versus technical replication
- The data obtained should be provided and sufficient information should be provided to indicate the number of independent biological and/or technical replicates
- If you encountered any outliers, you should describe how these were handled
- Criteria for exclusion/inclusion of data should be clearly stated
- High-throughput sequence data should be uploaded before submission, with a private link for reviewers provided (these are available from both GEO and ArrayExpress)

Please outline where this information can be found within the submission (e.g., sections or figure legends), or explain why this information doesn’t apply to your submission:

The data was collected in two to three week periods for each animal. Depending on the health of the animal and the quality of the neural recordings one to four experiments were performed per day. This information is included in Table Supplement 1. The criteria for including/excluding the data is explained in the methods section.

**Statistical reporting**

- Statistical analysis methods should be described and justified
- Raw data should be presented in figures whenever informative to do so (typically when N per group is less than 10)
- For each experiment, you should identify the statistical tests used, exact values of N, definitions of center, methods of multiple test correction, and dispersion and precision measures (e.g., mean, median, SD, SEM, confidence intervals; and, for the major substantive results, a measure of effect size (e.g., Pearson's r, Cohen's d)
- Report exact p-values wherever possible alongside the summary statistics and 95% confidence intervals. These should be reported for all key questions and not only when the p-value is less than 0.05.

Please outline where this information can be found within the submission (e.g., sections or figure legends), or explain why this information doesn’t apply to your submission:

The detailed description of the statistical analysis can be found in the methods and results sections. The p-values are reported in the results section. In addition we have provided an additional excel file, which includes the statistical methods, p-values and N for all of the analysis and related figures.

(For large datasets, or papers with a very large number of statistical tests, you may upload a single table file with tests, Ns, etc., with reference to sections in the manuscript.)

**Group allocation**

- Indicate how samples were allocated into experimental groups (in the case of clinical studies, please specify allocation to treatment method); if randomization was used, please also state if restricted randomization was applied
- Indicate if masking was used during group allocation, data collection and/or data analysis

Please outline where this information can be found within the submission (e.g., sections or figure legends), or explain why this information doesn’t apply to your submission:

Data were grouped in two ways. First, recordings were grouped as being from the primary or secondary brain area. These groups were defined by the timing of the evoked response, as described in the manuscript. Second, sessions were grouped based on stimulation protocol: the number of stimulation sites, the timing between stimulation at two sites, and control sessions in which no stimulation was applied. The complete explanation of these experimental groups can be found in both results and methods sections.

**Additional data files (“source data”)**

- We encourage you to upload relevant additional data files, such as numerical data that are represented as a graph in a figure, or as a summary table
- Where provided, these should be in the most useful format, and they can be uploaded as “Source data” files linked to a main figure or table
- Include model definition files including the full list of parameters used
- Include code used for data analysis (e.g., R, MatLab)
- Avoid stating that data files are “available upon request”

Please indicate the figures or tables for which source data files have been provided:

We have provided the numerical data (.mat format) for all of the graphs in all of the figures except where images or raw data were presented. For each figure we are providing “ReadMe” files that include descriptions of the parameters used. In addition the Matlab code for generating these figures are provided.
